# Effects of Amino Acid Side-Chain Length and Chemical Structure on Anionic Polyglutamic and Polyaspartic Acid Cellulose-Based Polyelectrolyte Brushes

**DOI:** 10.3390/polym13111789

**Published:** 2021-05-28

**Authors:** Dmitry Tolmachev, George Mamistvalov, Natalia Lukasheva, Sergey Larin, Mikko Karttunen

**Affiliations:** 1 Institute of Macromolecular Compounds, Russian Academy of Sciences, Bolshoy pr. 31, 199004 Petersburg, Russia; luk@imc.macro.ru (N.L.); selarin@macro.ru (S.L.); 2 Faculty of Physics, St. Petersburg State University, Petrodvorets, 198504 Petersburg, Russia; mamistvalov.georgii@gmail.com; 3 Department of Chemistry, The University of Western Ontario, 1151 Richmond Street, London, ON N6A 5B7, Canada; 4 Department of Applied Mathematics, The University of Western Ontario, 1151 Richmond Street, London, ON N6A 5B7, Canada; 5 The Centre of Advanced Materials and Biomaterials Research, The University of Western Ontario, 1151 Richmond Street, London, ON N6A 5B7, Canada

**Keywords:** mineralization, polyelectrolyte brushes, poly(amino acids), poly-(α,L-glutamic acid), poly-(α,L-aspartic acid), cellulose, molecular dynamics simulation

## Abstract

We used atomistic molecular dynamics (MD) simulations to study polyelectrolyte brushes based on anionic α,L-glutamic acid and α,L-aspartic acid grafted on cellulose in the presence of divalent CaCl_2_ salt at different concentrations. The motivation is to search for ways to control properties such as sorption capacity and the structural response of the brush to multivalent salts. For this detailed understanding of the role of side-chain length, the chemical structure and their interplay are required. It was found that in the case of glutamic acid oligomers, the longer side chains facilitate attractive interactions with the cellulose surface, which forces the grafted chains to lie down on the surface. The additional methylene group in the side chain enables side-chain rotation, enhancing this effect. On the other hand, the shorter and more restricted side chains of aspartic acid oligomers prevent attractive interactions to a large degree and push the grafted chains away from the surface. The difference in side-chain length also leads to differences in other properties of the brush in divalent salt solutions. At a low grafting density, the longer side chains of glutamic acid allow the adsorbed cations to be spatially distributed inside the brush resulting in a charge inversion. With an increase in grafting density, the difference in the total charge of the aspartic and glutamine brushes disappears, but new structural features appear. The longer sides allow for ion bridging between the grafted chains and the cellulose surface without a significant change in main-chain conformation. This leads to the brush structure being less sensitive to changes in salt concentration.

## 1. Introduction

It is well known that chemical modifications of surfaces of a material allow for tuning and controlling many of their properties and structure. One common modification is grafting polyelectrolyte molecules on the surface [1]. Conformations of the grafted polyelectrolyte molecules, or polyelectrolyte brush, depend on properties such as osmotic pressure of counterions, electrostatic repulsion, and steric interaction [2]. For example, brush-like structures can be used to provide a low friction coefficient for the development of new lubricant materials [3]. Moreover, the polyelectrolyte nature of the grafted chains makes brush structures tunable by external conditions, e.g., pH and ionic strength of the solution, electromagnetic field, or presence of multivalent salt, allowing for the design of stimuli-responsive materials. Adhesive properties of polyelectrolyte brushes make them usable for a wide range of applications, including water treatment [4], development of anticorrosion agents [5], antifouling materials [6], drug delivery [7] sensors [8], and tissue engineering materials [9].

The balance between two opposite factors determines the size of the brush: Osmotic pressure of counterions and elasticity of the grafted chains [10]. According to the theory for polyelectrolyte brushes in the presence of salt suggested by Zhulina et al. [10,11], increasing salt concentration leads to decreasing osmotic pressure and, consequently, the size of the brush reduces. In the case of multivalent counterions in solution, ion bridges between the charged groups of the polyelectrolyte start to play an important role in the formation of the brush structure. Brettmann et al. have shown that ion bridges between grafted chains lead to a significant reduction in the brush size [12]. Moreover, in their subsequent studies, Brettmann et al. demonstrated the formation of lateral irregularities in the brush structure caused to ion bridging—this has a significant effect on brush height [13,14].

Theories describing polyelectrolyte brush behavior do not consider the details of the brushes’ chemical structures, which determine rigidity and interactions between the grafted chains. In some cases, the chemical structure may play a significant role in the structure formation of the polymer brush. For example, Glova et al. discovered a nontrivial brush structure based on oligomers of lactic acid [15,16,17,18,19]: An interplay between chemical structure, persistence length, and dipole–dipole interactions between the residues of the grafted chains lead to the emergence of hairpin-like structures.

In this work, we used MD simulations to investigate the structure of brushes based on α,L-oligomers of anionic amino acids (glutamic and aspartic acid) grafted onto the surface of nanocrystalline cellulose immersed in water and with multivalent salt solutions. Anionic amino acids are promising for surface modifications due to their biocompatibility, biodegradability, and relative cheapness [20]. Cellulose is the most common polymer that is also biocompatible and biodegradable, and due to its complex hierarchical supramolecular structure, crystalline cellulose has unique properties: Cellulose nanofibrils and nanocrystals have excellent mechanical properties comparable to those of steel [21,22], making cellulose an excellent candidate for reinforcing polymeric materials [23,24]. The biological and physical properties discussed above make cellulose a versatile material for medical applications, particularly for wound dressings [25,26] and for diverse tissue scaffolds [27,28,29]. Materials based on cellulose modified by poly(anionic acids) are also used for the development of metal sorption membranes [30], equipment for virus-capturing [31], and bone scaffolds [9].

Despite a wide range of applications, the importance of choosing a particular type of anionic poly(amino acid) for modifications has not been properly addressed. Recent results show, however, that this issue requires further attention. For example, despite the very similar chemical structures of the α,L-oligomers of glutamic and aspartic acids (Figure S1a,b), it has been shown that they interact differently with multivalent salts [32,33,34], and Thula et al. have demonstrated a significant difference in organic matrix mineralization by various anionic poly(amino acids) [34]. Similarly, Picker et al. have shown that aspartic and glutamic acids have qualitatively different effects on calcium carbonate crystallization [33]. In our previous study, we demonstrated the formation of different structures of organomineral complexes by polyaspartic and polyglutamic acids in the presence of calcium salt solutions [32].

The differences in chemical structures can be the reason for the various brush structures taken by the different anionic poly(amino acids). The longer side chain of glutamic acid leads to a higher density of the grafted layer at the same degree of substitution. Consequently, the transition from the osmotic brush mode to the quasi-neutral mode (where steric interactions start to play a significant role) should occur at a lesser degree of surface modification for glutamic acid grafted chains. At lower grafting density, specific interactions between the grafted chains and cellulose play an important role in determining the brush structure; both amino acid and cellulose have active groups, which can be involved in specific interactions, H-bonds and dipole–dipole interactions in particular. These interactions, however, could be prevented by long charged amino acid side chains (the chemical structures are shown in Figure S1). Thus, the structure of the grafted interfacial layer remains unclear, yet knowing it is crucial for determining the material’s properties and response to changing conditions. Despite the prevalence of the investigated materials, the importance of choosing the current anionic amino acid for cellulose modification has not been discussed before. The aim of our work is to study the influence of grafted amino acid oligomers’ chemical structures on the structure and properties of cellulose-based brushes in both water and multivalent salt solution. Prediction of brush structures is challenging, and atomistic MD simulations provide a feasible method to study the issue and the physical origins of the different structures.

One particular difficulty concerning MD simulations is the presence of multivalent ions, as this can lead to strong interactions that can severely restrict conformational transitions. Several advanced techniques have been developed to overcome high energy barriers, including replica exchange [35], metadynamics [36], and its variants [37,38,39]. In our previous work [32], we performed Hamiltonian replica exchange simulations of anionic poly(amino acids) in CaCl_2_ solution and showed the emergence of calcium bridges between carboxyl groups in simulations of polyelectrolytes. This poses a severe obstacle to proper sampling. To increase sampling, many replicas are needed, which is extremely resource-demanding and makes a variation of the systems difficult. In this work, we used well-tempered metadynamics [38] and unbiased MD simulations to overcome such problems.

## 2. Models and Methods

### 2.1. Model Parameters

**Figure 1:**
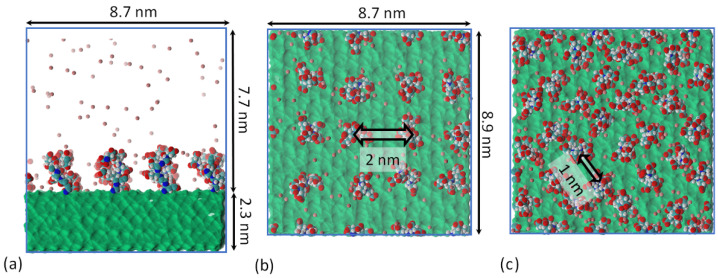
Grafted cellulose model. Side view of a model cellulose layer in a simulation box (**a**). Spatial distribution of grafted chains at 12% (**b**) and 25% (**c**) substitution. Green surface: cellulose crystal. Red: oxygen; cyan: carbons; white: hydrogens; blue: nitrogen; pink: K^+^ ions. Water molecules are not shown for clarity.

The same model for a cellulose layer was used as in our previous study of mineralization of phosphorylate cellulose [40]. The model for native cellulose was based on crystallographic data of cellulose Ib and the atomic coordinates of cellulose molecules in a crystal cell with parameters a = 0.82 nm, b = 0.78 nm, c = 1.038 nm, β = 90°, α = 90°, and γ = 96.6° [41]. The thickness of the layer was 2.3 nm (Figure 1). Cellulose molecules were considered to be infinite, connected through periodic boundary conditions. The layer consisted of 64 cellulose oligomers, each containing 16 glucose residues.

The cellulose surface was modified by replacing the primary hydroxyl groups attached to the rings through the methylene groups; the experimental procedure to perform this has been described in several studies [23,42,43,44,45]. Grafting was realized via the *N*-end of amino acid. The C-termini of the grafted chains were terminated by a carboxyl group. The degree of polymerization of the grafted chains was 6. This degree of polymerization corresponds to experimental studies of grafting of glutamic acid oligomers onto cellulose nanocrystals [23]. The use of short chains allows for high grafting density.

Two levels of substitution, 12% and 25%, of primary hydroxyl groups corresponding to grafting densities σ = 0.2 nm^−2^ and σ = 0.4 nm^−2^, were considered. The same degrees of cellulose surface modification were used in our previous work devoted to the investigation of the phosphorylated cellulose mineralization [40]. The choice of these modification degrees allowed us to investigate two regimes of the grafting densities: the charged mushroom regime and the osmotic regime. A preliminary simulation of a single grafted chain showed that the inverse surface area occupied by one isolated chain is σ* ≈ 0.3 nm^−2^. Thus, systems with σ = 0.2 nm^−2^ represent the charged mushroom regime (regime without steric interactions between grafted chains) and systems with σ = 0.4 nm^−2^ represent the osmotic regime [46]. All carboxyl groups were deprotonated, which corresponds to a solution with pH > 7. This choice is based on the results of Terauchi et al. [47], who have shown that with the increase of molecular weight, the pKa value increases and is 5.74 for poly(aspartic acid) and 6.05 for poly(glutamic acid). At pH 7.4, the degree of ionization for poly(aspartic acid) is 0.94 and 0.9 for poly(glutamic acid).

The initial dimensions of the simulation box were 8.7 nm × 8.9 nm × 10 nm. After creating a chemically modified cellulose model, the free space of the box was filled with water molecules. Part of the water molecules was randomly replaced by counterions to ensure overall electroneutrality. Due to the reported artificially strong attractions between Na+ ions and charged groups, which may cause unrealistic chain conformations [48,49,50], K^+^ ions were used as counterions. The systems and their compositions are shown in Table 1.

After obtaining an equilibrium brush structure in an aqueous solution (the criteria of the equilibration are discussed below), part of the water molecules was replaced with CaCl_2_ ions. Five concentrations representing a concentration range from partial neutralization of the brush charge to excessive ion adsorption were used; see Table 1. Figure 1 shows an example of a starting configuration with glutamic acid oligomers.

### 2.2. MD Simulation Parameters

All the simulations were performed with the Gromacs 2016.3 software [51]. For amino acids and ions, the CHARMM27 force field (CHARMM22/CMAP) [52] was used, and for cellulose CSFF (carbohydrate solution force field) [53] based on CHARMM22 was employed. This combination of force fields has been successfully used in our previous simulations of native and phosphorylated cellulose in salt solutions [40,54].

For ion–ion interactions, we used the correction proposed by Church et al. [55]. This approach is based on modifying the Lennard-Jones σ-parameter for the interactions between the Ca^2+^ ions and the carbonyl oxygens of the glutamic acid residues. This modification has been shown to produce a correct representation of the interaction energy corresponding to experimental NMR data [56,57].

For water molecules, the CHARMM compatible version of the TIP3P model was used [58]. All simulations were performed in the isothermal-isobaric (NPT) ensemble at *p* = 1 bar and T = 300 K. To maintain constant temperature and pressure, the Nosé-Hoover thermostat [59,60] and the Parrinello-Rahman barostat [61] were used. Electrostatic interactions were treated using the particle-mesh Ewald (PME) method [62]. The P-LINCS algorithm [63] was used to constrain bond lengths involving hydrogen atoms. Visualization of trajectories was performed using the Visual Molecular Dynamics (VMD) software [64].

Simulations of systems without salt were performed by unbiased MD. Each system was simulated for 500 ns with a 2 fs time step. The first 100 ns were considered as equilibration. Our previous studies have shown that this time is enough to achieve equilibrium [40,49] and it was also verified by measuring the distribution of K^+^ ions (Figure S2). After the above 100 ns equilibration followed by the addition of CaCl_2_, preliminary equilibration was performed by unbiased 20 ns MD simulations. The final configurations of these simulations were used as starting configurations for well-tempered metadynamics simulations [38].

### 2.3. Metadynamics Simulation Parameters

Well-tempered metadynamics simulations were performed by Gromacs software patched by PLUMED 2.4.2 [65]. One of the challenges in metadynamics is the determination of the correct collective variables (CV). Since interactions between Ca^2+^ ions and carboxyl groups play a key role in structure formation, we chose the number of the carboxyl groups free from adsorbed Ca^2+^ ions as the collective variable. Free carboxyl groups were defined via:

(1) $$s=\frac{1-{\left(\frac{r}{r_{0}}\right)}^{6}}{1-{\left(\frac{r}{r_{0}}\right)}^{12}}$$

where *r* is the distance between the carboxyl oxygen and a Ca^2+^ ion. The variable *r*_0_ is defined as the maximum of the radial distribution function between the oxygens of carboxyl groups and a Ca^2+^ ion (the radial distribution function is shown in Figure S3). If the function is equal to zero, the carboxyl group is considered to be free from Ca^2+^ ions. Metadynamics is based on the addition of Gaussians to the energy landscape using CVs. Here, the Gaussian width was set to 0.05 and the initial Gaussian deposition rate to 2 kJ/mol per ps, with a bias factor of 20. The changing of CV during the time simulation is shown in Figure S4. We have equilibrated for an additional 150 ns using metadynamics and used the time interval 150–450 ns as a production run for collecting data. A similar approach has been successfully used to simulate ion adsorption on phospholipid membranes [66] and the formation of calcium phosphate prenucleation clusters [67].

## 3. Results

### 3.1. Structure of Brush in Pure Water

**Figure 2:**
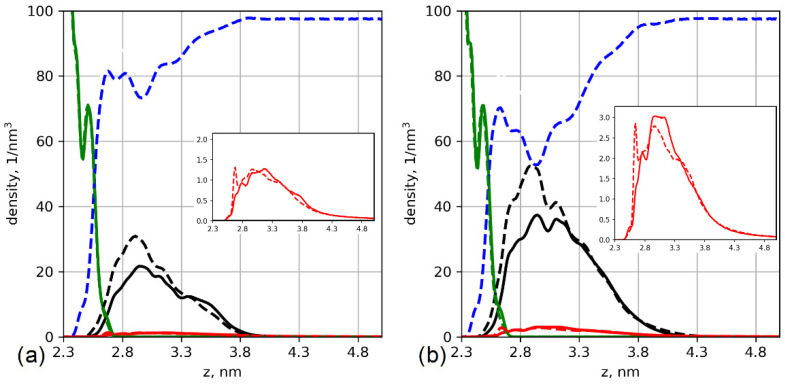
Number density profiles of the brush based on the oligomers of aspartic (solid lines) and glutamic (dashed lines) acids. The density profiles of cellulose, brush, water, and K^+^ ions are shown by green, black, blue, and red lines, respectively. (**a**) Systems with 12% substitution of primary hydroxyl groups and (**b**) systems with 25% substitution. Insets show K^+^ ion distributions on a larger scale for clarity.

The difference between aspartic and glutamic acid oligomers grafted onto the surface is already observed in the systems without salt. To investigate the effects of the side chain length on the structure of the grafted layer, we studied component-wise density profiles, Figure 2.

Figure 2 demonstrates the differences between the structures of the brushes based on the two different amino acids. The additional methylene group in the glutamic acid residue increases the total density of the grafting layer. The boundary of the glutamic acid brush density is slightly shifted toward the cellulose surface. This may result from the specific interactions between grafted chains and the surface—this is discussed in later sections of the paper. The differences in the distributions also lead to differences in hydration. Due to the higher density of the glutamic acid brush, the amount of water is lower and water is not evenly distributed inside the brush. Although the cellulose surface is well solvated, the amount of water decreases with increasing brush density. Counterion distribution also depends on the type of amino acid. In particular, K^+^ ion distribution in the glutamic acid brush has a pronounced peak close to the surface (see the insets in Figure 2).

**Figure 3:**
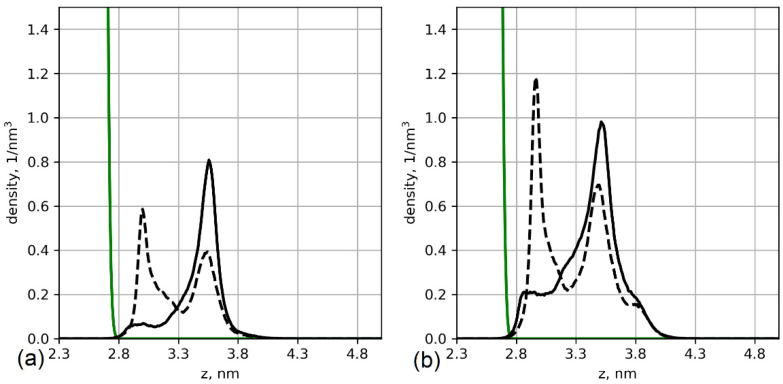
Number density profiles of the chain ends of the grafted oligomers of aspartic (solid lines) and glutamic (dashed lines) acids. (**a**) Systems with 12% substitution of primary hydroxyl groups and (**b**) systems with 25% substitution. The green line indicates the density of the cellulose layer.

To understand the origins of these effects, we analyzed the conformations of the grafted chains; Figure 3 shows the chain end distributions. The carbon of the methylene group of the main chain (Cα) of the last residue was chosen as the control atom for the analysis.

**Figure 4:**
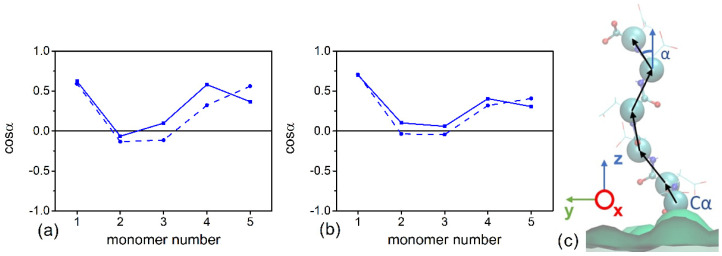
Average cosine of the angle between the vectors connecting Cα atoms of neighbor residues and the axis perpendicular to the surface. Solid lines: aspartic acid oligomers; dashed lines: glutamic acid oligomers. (**a**) Systems with 12% substitution of primary hydroxyl groups, (**b**) systems with 25% substitution, and (**c**) scheme illustrating the angles.

**Figure 5:**
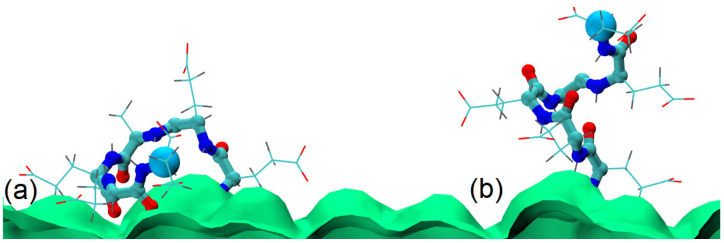
Illustrations of two typical conformations of a glutamic acid oligomer: (**a**) chain lying on the cellulose surface and (**b**) chain directed away from the surface. Cellulose surface is green, the side chains are illustrated by lines, CPK representation is used for the backbone, and Cα atoms of the last residue are visualized using a larger size.

Figure 3 shows that the ends of the glutamic acid oligomers have a bimodal distribution, while the chains of aspartic acid are mostly elongated. This can be explained by the differences in interactions with the cellulose layer. As the grafting density increases, the glutamic acid oligomers become more elongated, and their ends are located farther from the cellulose surface. Figure 4 shows the orientations of the chain residues in terms of cosine of the angle between the vectors connecting Cα atoms of neighbor monomers and the axis perpendicular to the surface. The orientation of the chain monomers confirms that a large number of glutamic acid oligomers lie on the cellulose surface. For the system with 12% substitution (Figure 4a), the average cosine is close to zero and even negative (for the second and third monomers), which indicates a vector directed towards the surface. With an increase in grafting density (Figure 4b), the average cosine value increases, indicating a change in the ratio of two states of the chains (lying on and directed from the surface). Snapshots from simulations are shown in Figure 5.

**Figure 6:**
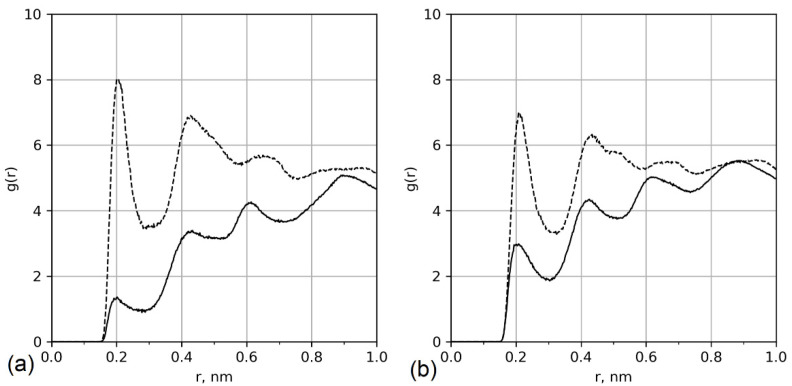
Radial distribution functions between the cellulose surface (hydrogens of surface hydroxyl groups) and amino acid backbone (O of amide groups). Solid lines: aspartic acid oligomers; dashed lines: glutamic acid oligomers. (**a**) Systems with 12% substitution of primary hydroxyl groups and (**b**) systems with 25% substitution.

The reason for chains lying on the surface is specific interactions between the amide groups of the main chain and the hydroxyl groups of the cellulose surface. This is demonstrated in Figure 6, which shows the radial distribution function between the oxygen atoms of the amide groups and the hydrogens of the hydroxyl groups. The radial distribution function was calculated as

(2) $$g_{AB}\left(r\right)=\frac{1}{N_{A}\rho_{B}}\sum_{i\subset A}^{N_{A}}\sum_{j\subset A}^{N_{B}}\frac{\delta \left(r_{ij}-r\right)}{4\pi r^{2}}$$

where *ρ_B_* is the average density of type *B* atoms around atoms *A*, *N_A_* and *N_B_* are the number of *A* and *B* atoms, respectively, *r_ij_* is the distance between two atoms *A* and *B*, and *δ* is the Kronecker delta function.

Systems with bimodal distributions of the grafted chain conformations (see Figure 3) are characterized by a peak in the radial distribution function for hydrogens of the surface hydroxyl groups and oxygens of the amino acid backbones at short distances (*r* = 0.2). This is typical for the H-bonds; however, a detailed analysis of H-bonds, including distance and angle criteria, showed that only 30% of the contacts are hydrogen bonds. The rest is the result of dipole–dipole interactions. In the case of a glutamic acid brush, only one H-bond binds the lying chain onto the surface (distributions of the number of close contacts between hydrogens of surface hydroxyl groups and oxygens of amide groups and H-bonds between them are shown in Figure S5).

**Figure 7:**
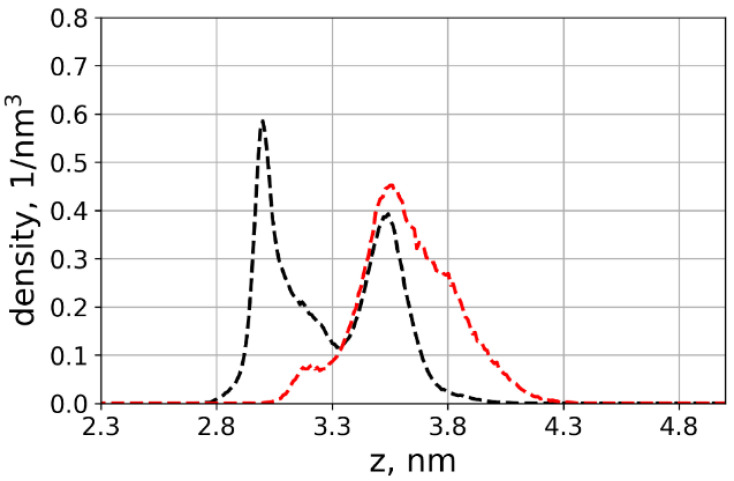
Density profiles of the chain ends of the grafted glutamic acid oligomers for the system with 12% substitution of primary hydroxyl groups by glutamic acid oligomers. Red line: System without partial charges on the cellulose molecules. Blue lines: with partial charges (unmodified force field).

To confirm that the chains indeed lie on the surface due to the interactions with the surface, we simulated an additional system in which we disabled partial atomic charges on the cellulose molecules. For this purpose, the system with the highest number of chains lying on the surface was chosen. The results and comparison with the systems with partial charges present are shown in Figure 7.

As the figure shows, the oligomers extend away from the cellulose surface when the partial charges are set to zero, i.e., dipole–dipole interactions and H-bonds cannot form. Thus, the absence of attractive interactions between the oligomer and the cellulose surface is the reason for the first peak in Figure 7 shifting to higher values. This result confirms that the reason for the bimodal distribution of the glutamic acid oligomer conformation shown in Figure 3 is H-bonding and dipole–dipole interactions between the grafted chain and cellulose surface.

Side-chain length is a major reason for the different behaviors. A longer side chain may prevent interactions with the surface; however, in the case of glutamic acid, the additional methylene group in the side chain gives it an additional degree of freedom, which makes it possible to rotate the side chains and allow for interactions between the main chains of the grafted molecules and the cellulose surface. Moreover, the additional group in the side chain helps to overcome the dihedral angle energy barriers and make the chain more flexible; the energy barrier for transitions between dihedral angles of aspartic acid is over kT higher than for glutamic acid. The potential of mean force obtained from the dihedral distribution is shown in Figure S6.

### 3.2. The Structure of the Brush in CaCl_2_ Solution

**Figure 8:**
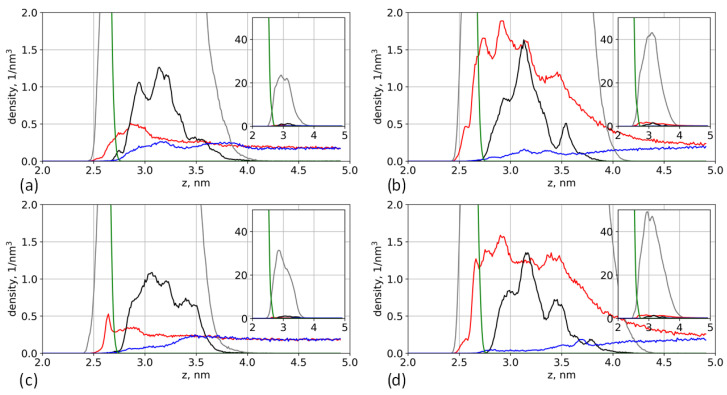
Number density profiles of the brush for (**a**,**b**) aspartic acid brushes and (**c**,**d**) glutamic acid brushes. The density profiles of cellulose, brush, Ca^2+^, K^+^, and Cl^−^ ions are shown by green, purple, black, red, and blue lines, respectively. Insets show close-ups of brush distributions. (**a**,**c**) Systems with 12% substitution of primary hydroxyl groups and (**b**,**d**) systems with 25% substitution. CaCl_2_ concentration is 0.15 mol/kg.

The addition of CaCl_2_ in the solution leads to changes in the brush structures. Figure 8 shows the typical distributions of the components at the CaCl_2_ concentration of 0.15 mol/kg.

**Figure 9:**
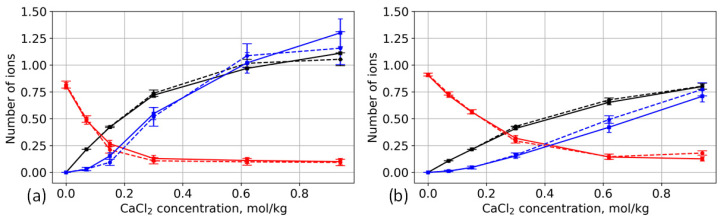
Dependence of the number of ions per carboxyl group (Ca^2+^, K^+^, Cl^−^) on CaCl_2_ concentration. Ca^2+^, K^+^, and Cl^−^ ions are shown by black, red, and blue lines, respectively. Solid lines: aspartic acid brushes; dashed lines: glutamic acid brushes. (**a**) Systems with 12% substitution of primary hydroxyl groups and (**b**) systems with 25% substitution.

Figure 8 shows that Ca^2+^ ions are adsorbed in the brush. Together with cations, Cl^−^ ions also penetrate the brush, partly compensating for the cation charge. Interestingly, the location of the Ca^2+^ ions differs from the location of K^+^ ions. A significant part of the K^+^ ions is located deeper inside the brush. This result correlates well with our previous work on mineralization of phosphorylated surfaces, where it was shown that Na^+^ ions are located deeper in the cellulose layer than Ca^2+^ ions [40]. The cellulose surface layer has a significantly lower dielectric constant, making the localization of ions energetically preferable; however, the sizes of ions with their respective hydration shells are too large to penetrate this layer. Unlike Ca^2+^ ions, monovalent ions can lose water from the hydration shell, allowing them to move deeper into the cellulose surface layer and establish ion–ion and ion–dipole interactions at a lower dielectric constant. Similar behavior has also been reported for lipid membranes [68]. Figure 9 shows the dependence of the number of ions (per one carboxyl group) inside the brush as a function of CaCl_2_ concentration.

**Figure 10:**
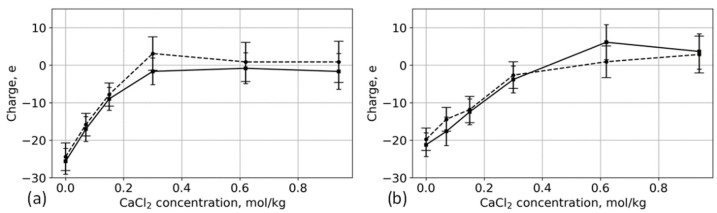
Total charge of the brush as a function of CaCl_2_ concentration. Solid lines: aspartic acid brushes; dashed lines: glutamic acid brushes. (**a**) Systems with 12% substitution of primary hydroxyl groups and (**b**) systems with 25% substitution.

As CaCl_2_ concentration increases, the divalent Ca^2+^ ions replace the monovalent K^+^ ones; however, the K^+^ ions located deeper in the layer remain there even at high concentration levels. In addition, as the number of the adsorbed Ca^2+^ ions increases, so does the number of Cl^−^ ions. As Figure 10 shows, at some concentrations, there is an overcompensation of charge inside the brush. It is important to mention that the sorption capacity of the brush per chain decreases as the degree of surface modification increases. The same dependence has been observed for phosphorylated cellulose in CaCl_2_ solution [40]. In the case of high grafting density, the adsorbed Ca^2+^ ions repel each other, and, consequently, they cannot occupy all vacant carboxyl groups.

Although there is no significant difference between the adsorption of individual types of ions by aspartic-acid-based and glutamic-acid-based brushes, the difference in the total charge of the brush is more distinguishable. Despite the fact that the absolute number of adsorbed ions at high concentrations is different for different degrees of surface modification, the ratio between the cations and anions gives a similar total charge. In the case of 12% substitution of hydroxyl groups, the total charge of the brushes reach saturation with the increase of the CaCl_2_ concentration starting from 0.3 mol/kg (Figure 10a); in the case of this low grafting density (the so-called charged mushroom mode), there is enough space inside the brush layer, and the longer side chains of glutamic acid are able to adsorb more ions. As the grafting density increases (transition to osmotic mode), the space becomes limited for both glutamic and aspartic acids, and the differences between brushes become indistinguishable (Figure 10b).

**Figure 11:**
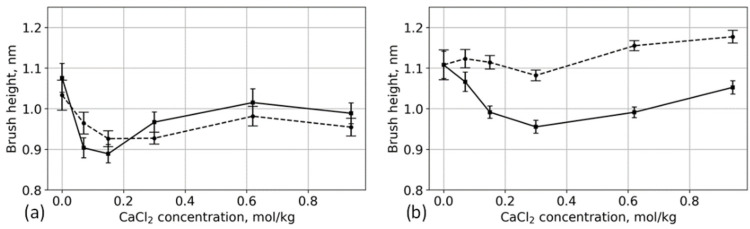
Dependence of the brush height on CaCl_2_ concentration. Solid lines: aspartic acid brushes; dashed lines: glutamic acid brushes. (**a**) Systems with 12% substitution of primary hydroxyl groups and (**b**) systems with 25% substitution.

The different side-chain lengths also affect the response to salt concentration. Figure 11 shows the brush height as a function of CaCl_2_ concentration. It was calculated using

(3) $$H=\frac{\int_{0}^{\infty }z\rho \left(z\right)dz}{\int_{0}^{\infty }\rho \left(z\right)dz}-z_{0},$$

where *ρ*(*z*) is the density profile of the brush along the z-coordinate and *z*_0_ is the coordinate of the grafting point.

At low grafting density (Figure 11a), the changes in the structure of the aspartic acid brush are more significant. The brush height decreases rapidly with the addition of salt at low concentrations of CaCl_2_ and partially recovers at higher concentrations. This behavior is similar to what has been reported for chain sizes of free polyelectrolytes in multivalent solutions [32,69,70]. Moreover, similar to the behavior of anionic poly (amino acid) chains [32], glutamic acid brushes remain folded over a wider concentration range than aspartic acid brushes. These similarities are associated with the low grafting density that, to a large degree, eliminates steric interactions.

As the grafting density increases (Figure 11b), the differences in the heights of the aspartic and glutamic acid brushes become more pronounced. The behavior of aspartic acid brush is qualitatively similar to the brush behaviors at low CaCl_2_ concentration (Figure 11a), the brush size decreases, and with a further increase in concentration, it is partly restored. This result is in qualitative agreement with the theoretical prediction for brush height versus concentration proposed by Brettmann et al. [12]. In the case of high grafting density, the concentration region of the collapsed aspartic acid brush is much wider due to the large number of carboxyl groups. The glutamic acid brushes at 25% surface modification show qualitatively different behavior: The glutamic acid brush is almost independent of salt concentration, and with an increase of salt concentration, the brush size increases. This result contradicts the theory [12], which does not predict an increase in brush height with the addition of a multivalent salt. This behavior is associated with the longer side chain, which can form calcium bridges without folding the chain, whereas the ideal polyelectrolyte (without side chains) considered by the theory does not have this ability.

**Figure 12:**
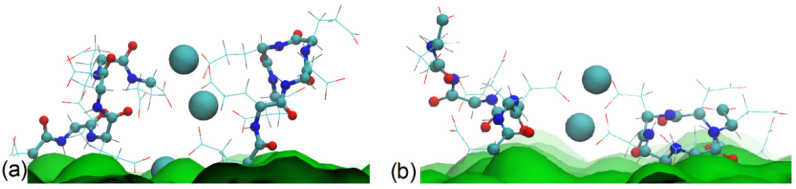
Snapshots illustrating Ca^2+^ bridges in (**a**) glutamic acid and (**b**) aspartic acid brushes.

Calcium ions in the brush tend to interact with two carboxyl groups connecting them and form long-living bridges [32]. At 25% surface modification, the distance between the grafting points is 1 nm (see Figure 1c), which is very close to the distance between the backbones of the grafted glutamic acid oligomers connected by a Ca^2+^ bridge. Thus, the formation of a Ca^2+^ bridge between adjacent grafted chains occurs practically without folding. With an increase in concentration, some of the surface-lying chains become directed away from the surface, trying to form the most energetically favorable intermolecular contacts with Ca^2+^ ions. This is reflected in the total height of the brush. The changes in the chain end distributions at different CaCl_2_ concentrations are shown in Figure S7. In the case of the aspartic acid brush, the formation of Ca^2+^ bridges between grafted chains brings them closer to each others’ main chains and decreases the brush size. Intermolecular Ca^2+^ bridges in aspartic acid and glutamic acid brushes are illustrated in Figure 12.

**Figure 13:**
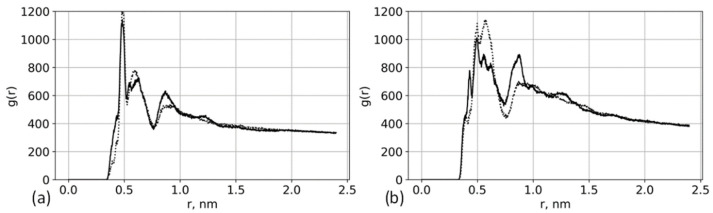
Ca^2+^-Ca^2+^ radial distribution functions for the systems with 0.94 mol/kg CaCl_2_ concentration. Solid lines: aspartic acid brushes; dashed lines: glutamic acid brushes. (**a**) Systems with 12% substitution of primary hydroxyl groups and (**b**) systems with 25% substitution.

Cellulose modified by anionic molecules is used for the synthesis of organomineral composites [9]. The distribution of Ca^2+^ ions and its dependence on brush structure is an important factor, which allows determining the structure for the formation of minerals in the brush [71,72]. To check how the differences between glutamic and aspartic brushes (Figure 11) influence the Ca^2+^ ion distributions, we analyzed the Ca^2+^-Ca^2+^ radial distribution functions for the systems at the highest considered CaCl_2_ concentration (Figure 13).

At lower grafting density (Figure 13a), the distribution of Ca^2+^ ions is practically the same, and at the higher grafting density (Figure 13b), some differences arise primarily in the peak intensities.

## 4. Conclusions

We have performed unbiased MD and well-tempered metadynamics simulations of polymer brushes based on two anionic amino acid oligomers, α,l-glutamic acid and α,l-aspartic acid, grafted onto cellulose surface in water and CaCl_2_ solutions. The results show that the structure and behavior of the polyelectrolyte brushes depend on the chain length, grafting density, and chemical structure.

In the case of the shorter aspartic acid oligomers, the side chains with the charged carboxyl groups prevent interactions with the cellulose surface, and they are pushed away from the surface. The glutamic acid oligomers show different behavior: their longer length and conformational freedom of the side chain due to the additional methylene group together with lower energy barriers between different states of the dihedral angles allow the side chains to rotate and to establish specific interactions with the cellulose surface. Due to this, the grafted chains display two populations: (1) chains lying on the surface of the cellulose and (2) chains directed away from the surface. This result is important from the point of view of chemical modifications. Chains lying on the surface can make it difficult to access the surface and any new free grafting points. This can become the limiting step that determines the maximum degree of chemical modifications of the cellulose surface. Moreover, the bimodal distribution of the chains leads to an uneven availability of end groups of the grafted chains. End group availability is important for the synthesis of copolymers on the cellulose surface or when using the “grafting from” approach [73], which consists of chain polymerization straight from the surface. In this case, the uneven availability of end groups leads to an uneven chemical modification of the surface.

The differences in the side chains also lead to a difference in brush structures in CaCl_2_ solutions. In the case of low grafting density at which the grafted chains do not interact by steric interactions, the behavior of the chains is similar to the behavior of polyelectrolyte chains in a multivalent salt [32,69,70]. The longer side chain of glutamic acid helps to spatially distribute the adsorbed ions inside the brush. This has an important effect on the total charge of the brush: the glutamic acid brush displays charge inversion; however, as the grafting density increases, the length of the side chain ceases to play a significant role in the total charge of the brush due to the lesser free volume in it.

At high grafting density, the presence of longer side chains makes the brush less sensitive to the environment. The grafted chains are able to adsorb ions and form intermolecular Ca^2+^ bridges. This allows the brush to absorb Ca^2+^ ions without significant changes in the brush height. Moreover, we have shown that the brush height increases with the addition of salt due to changes in the bimodal chain end bimodal distribution, as discussed above. Although the brush structures based on the two different amino acids are different, the distribution of mineral ions in them is similar, suggesting that both brushes should induce mineralization with a similar structure. It is plausible that the results of this investigation of the response of the brush structure on the presence of CaCl_2_ salt may be treated as a guide for the design of cellulose-based membranes for water purification and the development of mineral composites for tissue engineering.

## Figures and Tables

**Table 1:** List of brush structures considered in the simulations.

**Brush Structures Considered in the Simulation**				
**№**	**Amino Acid**	**Degree of Primary Hydroxyl Group Substitution, % (Grafting Density, 1/nm^2^)**	**Number of Grafted Chains**	**Number of K^+^ Ions**
1	Glutamic acid	12% (0.2)	16	112
2		25% (0.4)	32	224
3	Aspartic acid	12% (0.2)	16	112
4		25% (0.4)	32	224
**CaCl_2_ Concentrations Considered in the Simulations**				
**№**	**Concentration of CaCl_2_, mol/kg**	**Number of Ca^2+^ Ions**	**Number of Cl^−^ Ions**	
1	0	0	0	
2	0.07	24	48	
3	0.15	48	96	
4	0.30	96	192	
5	0.62	192	384	
6	0.94	288	576	

## Supplementary Materials

The following are available online at https://www.mdpi.com/article/10.3390/polym13111789/s1. Figure S1: Chemical structures of aspartic acid, glutamic acid, and cellulose residues. Figure S2: The number of K^+^ ions in the brush during the first 10 ns in the simulations without CaCl_2_ salt. Figure S3: Radial distribution function of Ca^2+^ ions around oxygens of amino acid carboxyl groups. Figure S4: Time dependence of the collective variables (carboxyl group free from adsorbed Ca^2+^ ions). Figure S5: Distributions of the number of close contacts between hydrogens of surface hydroxyl groups and oxygens of amide groups and H-bonds between them. Figure S6: Dihedral angle distributions of the side chain and potential of mean force obtained from the distributions. Figure S7: Density profiles of the ends of the grafted oligomers of aspartic and glutamic acids at different CaCl_2_ concentrations.

## References

[1] Das S., Banik M., Chen G., Sinha S., Mukherjee R. (2015). Polyelectrolyte brushes: Theory, modelling, synthesis and applications. Soft Matter.

[2] Chen W.-L., Cordero R., Tran H., Ober C.K. (2017). 50th Anniversary Perspective: Polymer Brushes: Novel Surfaces for Future Materials. Macromolecules.

[3] Xu X., Billing M., Ruths M., Klok H.-A., Yu J. (2018). Structure and Functionality of Polyelectrolyte Brushes: A Surface Force Perspective. Chem. Asian J..

[4] Wilts E.M., Herzberger J., Long T.E. (2018). Addressing water scarcity: Cationic polyelectrolytes in water treatment and purification. Polym. Int..

[5] Migahed M., Rashwan S., Kamel M., Habib R. (2017). Synthesized polyaspartic acid derivatives as corrosion and scale inhibitors in desalination operations. Cogent Eng..

[6] Jiang T., Yu X., Carbone E.J., Nelson C., Kan H.M., Lo K.W.-H. (2014). Poly aspartic acid peptide-linked PLGA based nanoscale particles: Potential for bone-targeting drug delivery applications. Int. J. Pharm..

[7] Sattari S., Tehrani A.D., Adeli M. (2018). pH-Responsive Hybrid Hydrogels as Antibacterial and Drug Delivery Systems. Polymers.

[8] Boyaciyan D., Krause P., Von Klitzing R. (2018). Making strong polyelectrolyte brushes pH-sensitive by incorporation of gold nanoparticles. Soft Matter.

[9] Stepanova M., Averianov I., Serdobintsev M., Gofman I., Blum N., Semenova N., Nashchekina Y., Vinogradova T., Korzhikov-Vlakh V., Karttunen M. (2019). PGlu-Modified Nanocrystalline Cellulose Improves Mechanical Properties, Biocompatibility, and Mineralization of Polyester-Based Composites. Materuals.

[10] Zhulina E.B., Birshtein T.M., Borisov O.V. (1995). Theory of Ionizable Polymer Brushes. Macromolecules.

[11] Zhulina E.B., Borisov O.V., Birshtein T.M. (1999). Polyelectrolyte Brush Interaction with Multivalent Ions. Macromolecules.

[12] Brettmann B.K., Laugel N., Hoffmann N., Pincus P., Tirrell M. (2015). Bridging contributions to polyelectrolyte brush collapse in multivalent salt solutions. J. Polym. Sci. Part A Polym. Chem..

[13] Yu J., Jackson N.E., Xu X., Brettmann B.K., Ruths M., De Pablo J.J., Tirrell M. (2017). Multivalent ions induce lateral structural inhomogeneities in polyelectrolyte brushes. Sci. Adv..

[14] Brettmann B., Pincus P., Tirrell M. (2017). Lateral Structure Formation in Polyelectrolyte Brushes Induced by Multivalent Ions. Macromolecules.

[15] Birshtein T., Polotsky A., Glova A., Amoskov V., Mercurieva A., Nazarychev V., Lyulin S. (2018). How to fold back grafted chains in dipolar brushes. Polymers.

[16] Glova A., Falkovich S.G., Larin S.V., Mezhenskaia D.A., Lukasheva N.V., Nazarychev V.M., Tolmachev D.A., Mercurieva A.A., Kenny J.M., Lyulin S.V. (2016). Poly(lactic acid)-based nanocomposites filled with cellulose nanocrystals with modified surface: All-atom molecular dynamics simulations. Polym. Int..

[17] Glova A.D., Larin S.V., Falkovich S.G., Nazarychev V.M., Tolmachev D.A., Lukasheva N.V., Lyulin S.V. (2017). Molecular dynamics simulations of oligoester brushes: The origin of unusual conformations. Soft Matter.

[18] Glova A.D., Larin S.V., Nazarychev V.M., Karttunen M., Lyulin S.V. (2019). Grafted Dipolar Chains: Dipoles and Restricted Freedom Lead to Unexpected Hairpins. Macromolecules.

[19] Mikhailov I., Amoskov V., Darinskii A., Birshtein T. (2020). The Structure of Dipolar Polymer Brushes and Their Interaction in the Melt. Impact of Chain Stiffness. Polymers.

[20] Thombre S.M., Sarwade B.D. (2005). Synthesis and Biodegradability of Polyaspartic Acid: A Critical Review. J. Macromol. Sci. Part A.

[21] Xie H., Du H., Yang X., Si C. (2018). Recent Strategies in Preparation of Cellulose Nanocrystals and Cellulose Nanofibrils Derived from Raw Cellulose Materials. Int. J. Polym. Sci..

[22] Wang W., Sabo R.C., Mozuch M.D., Kersten P., Zhu J.Y., Jin Y. (2015). Physical and Mechanical Properties of Cellulose Nanofibril Films from Bleached Eucalyptus Pulp by Endoglucanase Treatment and Microfluidization. J. Polym. Environ..

[23] Averianov I., Stepanova M.A., Gofman I.V., Nikolaeva A.L., Korzhikov-Vlakh V.A., Karttunen M., Korzhikova-Vlakh E.G. (2019). Chemical modification of nanocrystalline cellulose for improved interfacial compatibility with poly(lactic acid). Mendeleev Commun..

[24] Mao H., Wei C., Gong Y., Wang S., Ding W. (2019). Mechanical and Water-Resistant Properties of Eco-Friendly Chitosan Membrane Reinforced with Cellulose Nanocrystals. Polymers.

[25] Fürsatz M., Skog M., Sivlér P., Palm E., Aronsson C., Skallberg A., Greczynski G., Khalaf H., Bengtsson T., Aili D. (2017). Functionalization of bacterial cellulose wound dressings with the antimicrobial peptide ε-poly-L-Lysine. Biomed. Mater..

[26] Sulaeva I., Henniges U., Rosenau T., Potthast A. (2015). Bacterial cellulose as a material for wound treatment: Properties and modifications. A review. Biotechnol. Adv..

[27] Saska S., Barud H.S., Gaspar A.M.M., Marchetto R., Ribeiro S.J.L., Messaddeq Y. (2011). Bacterial Cellulose-Hydroxyapatite Nanocomposites for Bone Regeneration. Int. J. Biomater..

[28] Baklagina Y.G., Lukasheva N.V., Khripunov A.K., Klechkovskaya V.V., Arkharova N.A., Romanov D.P., Tolmachev D.A. (2010). Interaction between nanosized crystalline components of a composite based on Acetobacter xylinum cellulose and calcium phosphates. Polym. Sci. Ser. A.

[29] Buyanov A., Gofman I., Saprykina N. (2019). High-strength cellulose–polyacrylamide hydrogels: Mechanical behavior and structure depending on the type of cellulose. J. Mech. Behav. Biomed. Mater..

[30] Hestekin J.A., Bachas A.L.G., Bhattacharyya D. (2001). Poly(amino acid)-Functionalized Cellulosic Membranes: Metal Sorption Mechanisms and Results. Ind. Eng. Chem. Res..

[31] Sun M., Wang H., Li X. (2020). Modification of cellulose microfibers by polyglutamic acid and mesoporous silica nanoparticles for Enterovirus 71 adsorption. Mater. Lett..

[32] Tolmachev D., Lukasheva N., Mamistvalov G., Karttunen M. (2020). Influence of Calcium Binding on Conformations and Motions of Anionic Polyamino Acids. Effect of Side Chain Length. Polymers.

[33] Picker A., Kellermeier M., Seto J., Gebauer D., Cölfen H. (2012). The multiple effects of amino acids on the early stages of calcium carbonate crystallization. Z. Kristallogr. Cryst. Mat..

[34] Thula T.T., Svedlund F., Rodriguez D.E., Podschun J., Pendi L., Gower L.B. (2010). Mimicking the Nanostructure of Bone: Comparison of Polymeric Process-Directing Agents. Polymers.

[35] Sugita Y., Kamiya M., Oshima H., Re S. (2019). Replica-Exchange Methods for Biomolecular Simulations. Methods Mol. Biol..

[36] Barducci A., Bonomi M., Parrinello M. (2011). Metadynamics. Wiley Interdiscip. Rev. Comput. Mol. Sci..

[37] Meli M., Colombo G. (2013). A Hamiltonian Replica Exchange Molecular Dynamics (MD) Method for the Study of Folding, Based on the Analysis of the Stabilization Determinants of Proteins. Int. J. Mol. Sci..

[38] Barducci A., Bussi G., Parrinello M. (2008). Well-Tempered Metadynamics: A Smoothly Converging and Tunable Free-Energy Method. Phys. Rev. Lett..

[39] Galvelis R., Sugita Y. (2015). Replica state exchange metadynamics for improving the convergence of free energy estimates. J. Comput. Chem..

[40] Lukasheva N.V., Tolmachev D.A., Karttunen M. (2019). Mineralization of phosphorylated cellulose: Crucial role of surface structure and monovalent ions for optimizing calcium content. Phys. Chem. Chem. Phys..

[41] Nishiyama Y., Langan P., Chanzy H. (2002). Crystal Structure and Hydrogen-Bonding System in Cellulose Iβ from Synchrotron X-ray and Neutron Fiber Diffraction. J. Am. Chem. Soc..

[42] Majoinen J., Walther A., McKee J.R., Kontturi E., Aseyev V., Malho J.M., Ruokolainen J., Ikkala O. (2011). Polyelectrolyte Brushes Grafted from Cellulose Nanocrystals Using Cu-Mediated Surface-Initiated Controlled Radical Polymerization. Biomacromolecules.

[43] Li M., Liu Z., Wang L., James T.D., Xiao H.-N., Zhu W.-H. (2017). A glutamic acid-modified cellulose fibrous composite used for the adsorption of heavy metal ions from single and binary solutions. Mater. Chem. Front..

[44] Kang H., Liu R., Huang Y. (2015). Graft modification of cellulose: Methods, properties and applications. Polymers.

[45] Abushammala H., Mao J. (2019). A Review of the Surface Modification of Cellulose and Nanocellulose Using Aliphatic and Aromatic Mono- and Di-Isocyanates. Molcules.

[46] Borisov O.V., Birshtein T.M., Zhulina E.B. (1991). Collapse of grafted polyelectrolyte layer. J. Phys. II.

[47] Terauchi M., Tamura A., Tonegawa A., Yamaguchi S., Yoda T., Yui N. (2019). Polyelectrolyte Complexes between Polycarboxylates and BMP-2 for Enhancing Osteogenic Differentiation: Effect of Chemical Structure of Polycarboxylates. Polymers.

[48] Melcr J., Martinez-Seara H., Nencini R., Kolafa J., Jungwirth P., Ollila O.H.S. (2018). Accurate Binding of Sodium and Calcium to a POPC Bilayer by Effective Inclusion of Electronic Polarization. J. Phys. Chem. B.

[49] Tolmachev D.A., Boyko O.S., Lukasheva N.V., Martinez-Seara H., Karttunen M. (2019). Overbinding and Qualitative and Quantitative Changes Caused by Simple Na+ and K+ Ions in Polyelectrolyte Simulations: Comparison of Force Fields with and without NBFIX and ECC Corrections. J. Chem. Theory Comput..

[50] Venable R.M., Luo Y., Gawrisch K., Roux B., Pastor R.W. (2013). Simulations of Anionic Lipid Membranes: Development of Interaction-Specific Ion Parameters and Validation Using NMR Data. J. Phys. Chem. B.

[51] Abraham M.J., Murtola T., Schulz R., Páll S., Smith J.C., Hess B., Lindahl E. (2015). GROMACS: High performance molecular simulations through multi-level parallelism from laptops to supercomputers. SoftwareX.

[52] Mackerell A.D., Feig M., Brooks C.L. (2004). Extending the treatment of backbone energetics in protein force fields: Limitations of gas-phase quantum mechanics in reproducing protein conformational distributions in molecular dynamics simulations. J. Comput. Chem..

[53] Kuttel M., Brady J.W., Naidoo K.J. (2002). Carbohydrate solution simulations: Producing a force field with experimentally consistent primary alcohol rotational frequencies and populations. J. Comput. Chem..

[54] Lukasheva N.V., Tolmachev D.A. (2015). Cellulose Nanofibrils and Mechanism of their Mineralization in Biomimetic Synthesis of Hydroxyapatite/Native Bacterial Cellulose Nanocomposites: Molecular Dynamics Simulations. Langmuir.

[55] Church A.T., Hughes Z.E., Walsh T.R. (2015). Improving the description of interactions between Ca^2+^ and protein carboxylate groups, including γ-carboxyglutamic acid: Revised CHARMM22* parameters. RSC Adv..

[56] Daniele P.G., Foti C., Gianguzza A., Prenesti E., Sammartano S. (2008). Weak alkali and alkaline earth metal complexes of low molecular weight ligands in aqueous solution. Coord. Chem. Rev..

[57] Prorok M., Castellino F.J. (1998). Thermodynamics of Binding of Calcium, Magnesium, and Zinc to theN-Methyl-d-aspartate Receptor Ion Channel Peptidic Inhibitors, Conantokin-G and Conantokin-T. J. Biol. Chem..

[58] MacKerell A.D., Bashford D., Bellott M., Dunbrack R.L., Evanseck J.D., Field M.J., Fischer S., Gao J., Guo H., Ha S. (1998). All-Atom Empirical Potential for Molecular Modeling and Dynamics Studies of Proteins. J. Phys. Chem. B.

[59] Hoover W.G. (1985). Canonical dynamics: Equilibrium phase-space distributions. Phys. Rev. A.

[60] Nosé S. (1984). A molecular dynamics method for simulations in the canonical ensemble. Mol. Phys..

[61] Parrinello M., Rahman A. (1981). Polymorphic transitions in single crystals: A new molecular dynamics method. J. Appl. Phys..

[62] Darden T., York D., Pedersen L. (1993). Particle mesh Ewald: An *N*⋅log(*N*) method for Ewald sums in large systems. J. Chem. Phys..

[63] Hess B. (2008). P-LINCS: A Parallel Linear Constraint Solver for Molecular Simulation. J. Chem. Theory Comput..

[64] Humphrey W., Dalke A., Schulten K. (1996). VMD: Visual molecular dynamics. J. Mol. Graph..

[65] Tribello G.A., Bonomi M., Branduardi D., Camilloni C., Bussi G. (2014). PLUMED 2: New feathers for an old bird. Comput. Phys. Commun..

[66] Martí J. (2018). Free-energy surfaces of ionic adsorption in cholesterol-free and cholesterol-rich phospholipid membranes. Mol. Simul..

[67] Garcia N.A., Malini R.I., Freeman C.L., Demichelis R., Raiteri P., Sommerdijk N.A.J.M., Harding J.H., Gale J.D. (2019). Simulation of Calcium Phosphate Prenucleation Clusters in Aqueous Solution: Association beyond Ion Pairing. Cryst. Growth Des..

[68] Pöyry S., Róg T., Karttunen M., Vattulainen I. (2009). Mitochondrial Membranes with Mono- and Divalent Salt: Changes Induced by Salt Ions on Structure and Dynamics. J. Phys. Chem. B.

[69] Kundagrami A., Muthukumar M. (2008). Theory of competitive counterion adsorption on flexible polyelectrolytes: Divalent salts. J. Chem. Phys..

[70] Wei Y.-F., Hsiao P.-Y. (2010). Effect of chain stiffness on ion distributions around a polyelectrolyte in multivalent salt solutions. J. Chem. Phys..

[71] Grohe B., Hug S., Langdon A., Jalkanen J., Rogers K.A., Goldberg H.A., Karttunen M., Hunter G.K. (2012). Mimicking the Biomolecular Control of Calcium Oxalate Monohydrate Crystal Growth: Effect of Contiguous Glutamic Acids. Langmuir.

[72] Kahlen J., Peter C., Donadio D. (2015). Molecular simulation of oligo-glutamates in a calcium-rich aqueous solution: Insights into peptide-induced polymorph selection. CrystEngComm.

[73] Minko S., Stamm M. (2008). Grafting on solid surfaces: “Grafting to” and “grafting from” methods. Polymer Surfaces and Interfaces—Characterization, Modification and Applications.

