# Supplementary material for: Effects of Amino Acid Side-Chain Length and Chemical Structure on Anionic Polyglutamic and Polyaspartic Acid Cellulose-Based Polyelectrolyte Brushes
Source: Polymers (Basel). 2021 May 28;13(11):1789. doi: 10.3390/polym13111789 (PMC8199235; doi:10.3390/polym13111789)
Supplement: Supplementary file 1 [file polymers-13-01789-s001.zip › polymers-1234931-SI.pdf]

polymers

MDPI

Article

Effects of Amino Acid Side-Chain Length and Chemical Structure on Anionic Polyglutamic and Polyaspartic Acid Cellulose-Based Polyelectrolyte Brushes

Dmitry Tolmachev [1,*], George Mamistvalov [2], Natalia Lukasheva [1], Sergey Larin [1,2] and Mikko Karttunen [1,3,4,5,*]

[1] Institute of Macromolecular Compounds, Russian Academy of Sciences, Bolshoy pr. 31, 199004 Petersburg, Russia; luk@imc.macro.ru (N.L.); selarin@macro.ru (S.L.)
[2] Faculty of Physics, St. Petersburg State University, Petrodvorets, 198504 Petersburg, Russia; mamistvalov.georgii@gmail.com
[3] Department of Chemistry, The University of Western Ontario, 1151 Richmond Street, London, ON N6A 5B7, Canada
[4] Department of Applied Mathematics, The University of Western Ontario, 1151 Richmond Street, London, ON N6A 5B7, Canada
[5] The Centre of Advanced Materials and Biomaterials Research, The University of Western Ontario, 1151 Richmond Street, London, ON N6A 5B7, Canada
* Correspondence: dm.tolmahev@yandex.ru (D.T.); mkarttu@uwo.ca (M.K.)

Supplementary Informations

Citation: Tolmachev, D.; Mamistvalov, G.; Lukasheva, N.; Larin, S.; Karttunen, M. Effects of Amino Acid Side-Chain Length and Chemical Structure on Anionic Polyglutamic and Polyaspartic Acid Cellulose-Based Polyelectrolyte Brushes. *Polymers* **2021**, *13*, 1789. https://doi.org/10.3390/polym13111789

Academic Editor: Ranjit De

Received: 9 May 2021
Accepted: 25 May 2021
Published: 28 May 2021

Publisher's Note: MDPI stays neutral with regard to jurisdictional claims in published maps and institutional affiliations.

Copyright: © 2021 by the authors. Licensee MDPI, Basel, Switzerland. This article is an open access article distributed under the terms and conditions of the Creative Commons Attribution (CC BY) license (http://creativecommons.org/licenses/by/4.0/).

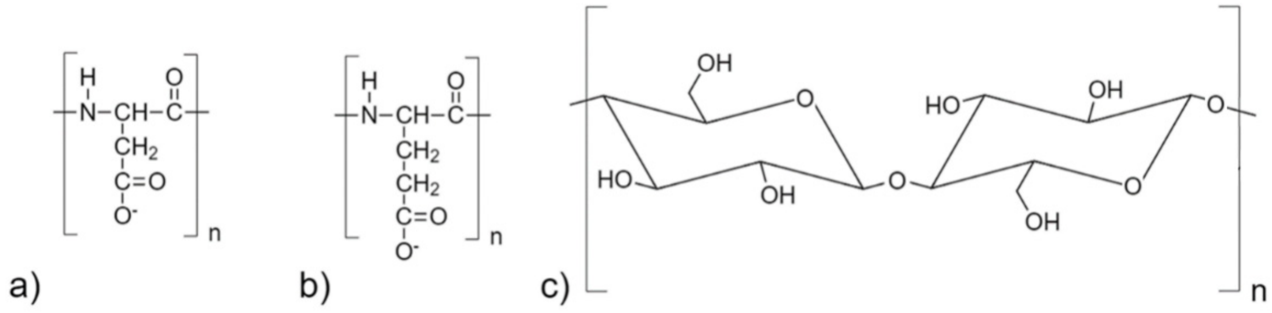

Figure S1. Chemical structures of a) aspartic acid, b) glutamic acid and c) cellulose residues. The degree of polymerization is denoted by n.

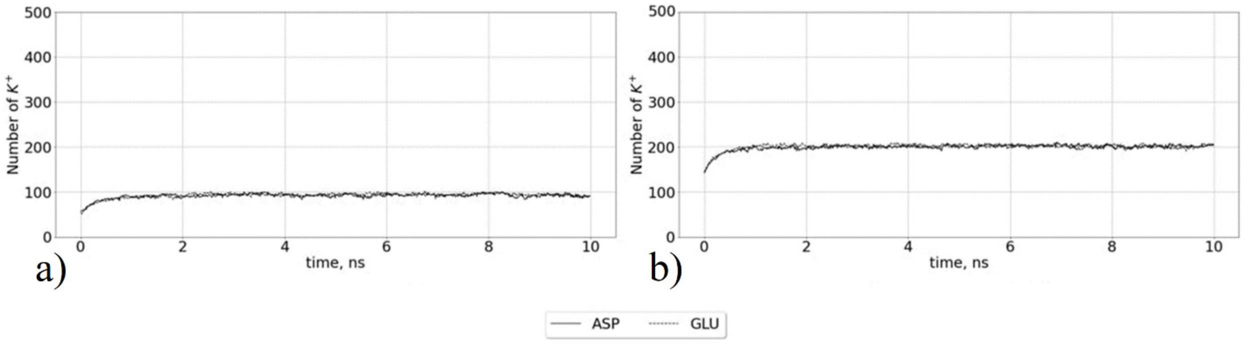

Figure S2. The number of K^+ ions in the brush during the first 10 ns in the simulations without $CaCl_2$ salt. a) systems with 12% degree surface modification, b) systems with 25% degree surface modification.

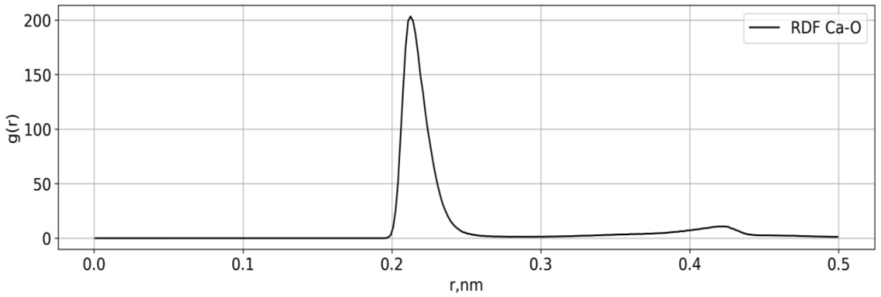

Figure S3. Radial distribution function of Ca^{2+} ions around oxygens of amino acid carboxyl groups.

Radial distribution function was calculated as:

$$g_{AB}(r) = \frac{1}{N_A \rho_B} \sum_{i \subset A}^{N_A} \sum_{j \subset A}^{N_B} \frac{\delta(r_{ij} - r)}{4\pi r^2} \quad (1)$$

where ρ_B is the average density of type B atoms around atoms A, N_A and N_B are the number of A and B atoms, respectively, r_{ij} is the distance between two atoms A and B, and δ is the Kronecker delta function.

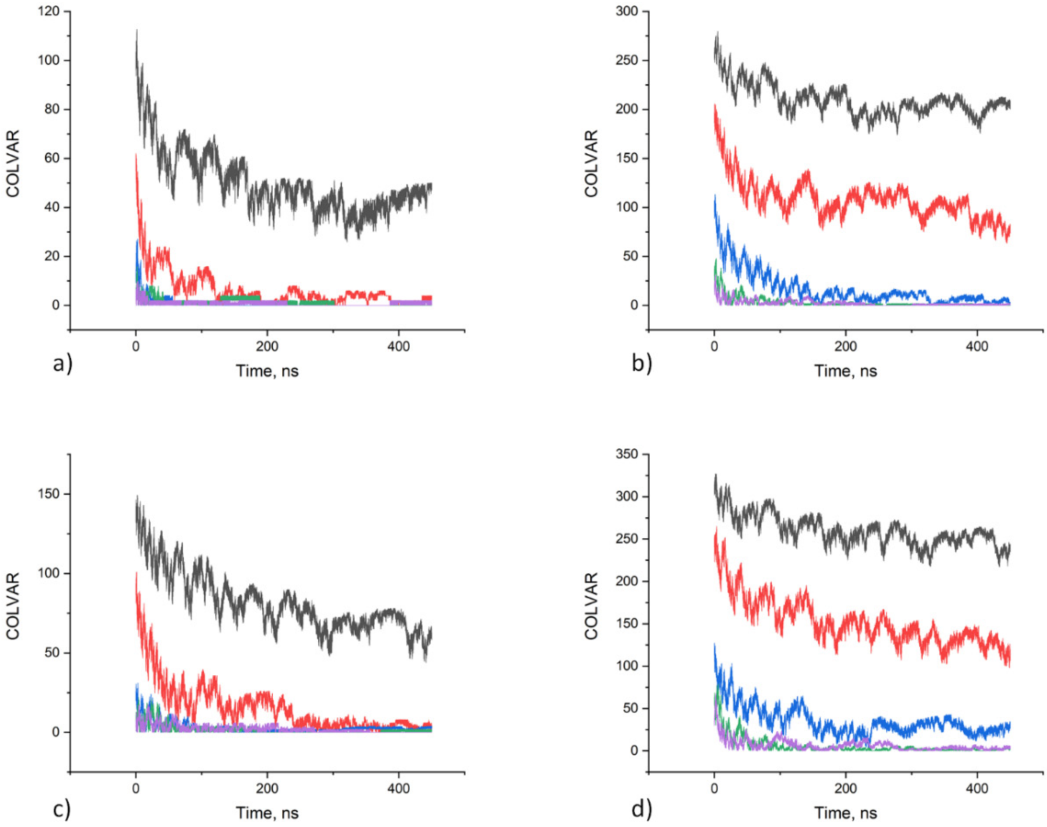

Figure S4. Time dependence of the collective variables (carboxyl group free from adsorbed calcium ions). The collective variable was calculated using differentiable switching function (1) in the text. The results of the simulation in different $CaCl_2$ concentration 0.07 mol/kg, 0.15 mol/kg, 0.30 mol/kg, 0.62 mol/kg, 0.94 mol/kg are presented by black, red, blue, green and purple colors correspondingly. a) Aspartic acid brush with 12% surface modification, b) aspartic acid brush with 25% degree surface modification, c) glutamic acid brush with 12% degree surface modification d) glutamic acid brush with 25% degree surface modification.

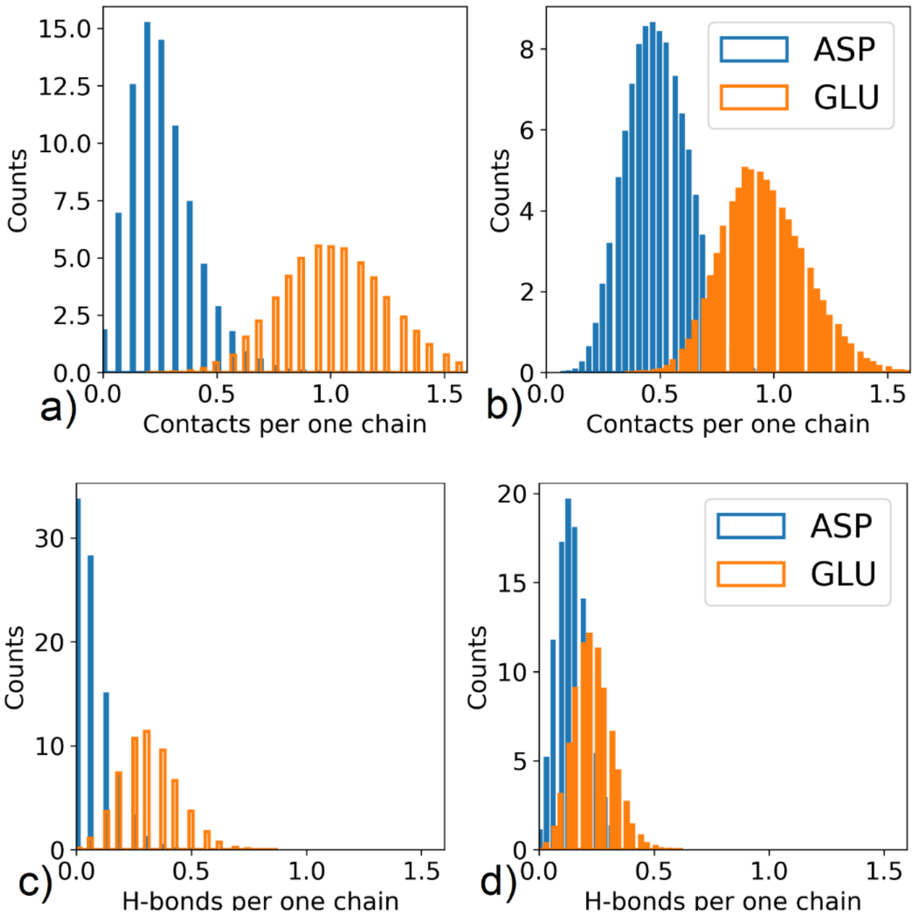

Figure S5. Distributions of the number of a, b) close contacts between hydrogens of surface hydroxyl groups and oxygens of amide groups and (the distance between atoms less then 0.35 nm) c,d) H-bonds between them (the distance between atoms less then 0.35 nm and the angle formed by acceptor, hydrogen and donor less than 35°). a,c) systems with 12% degree surface modification, b,d) systems with 25% degree surface modification.

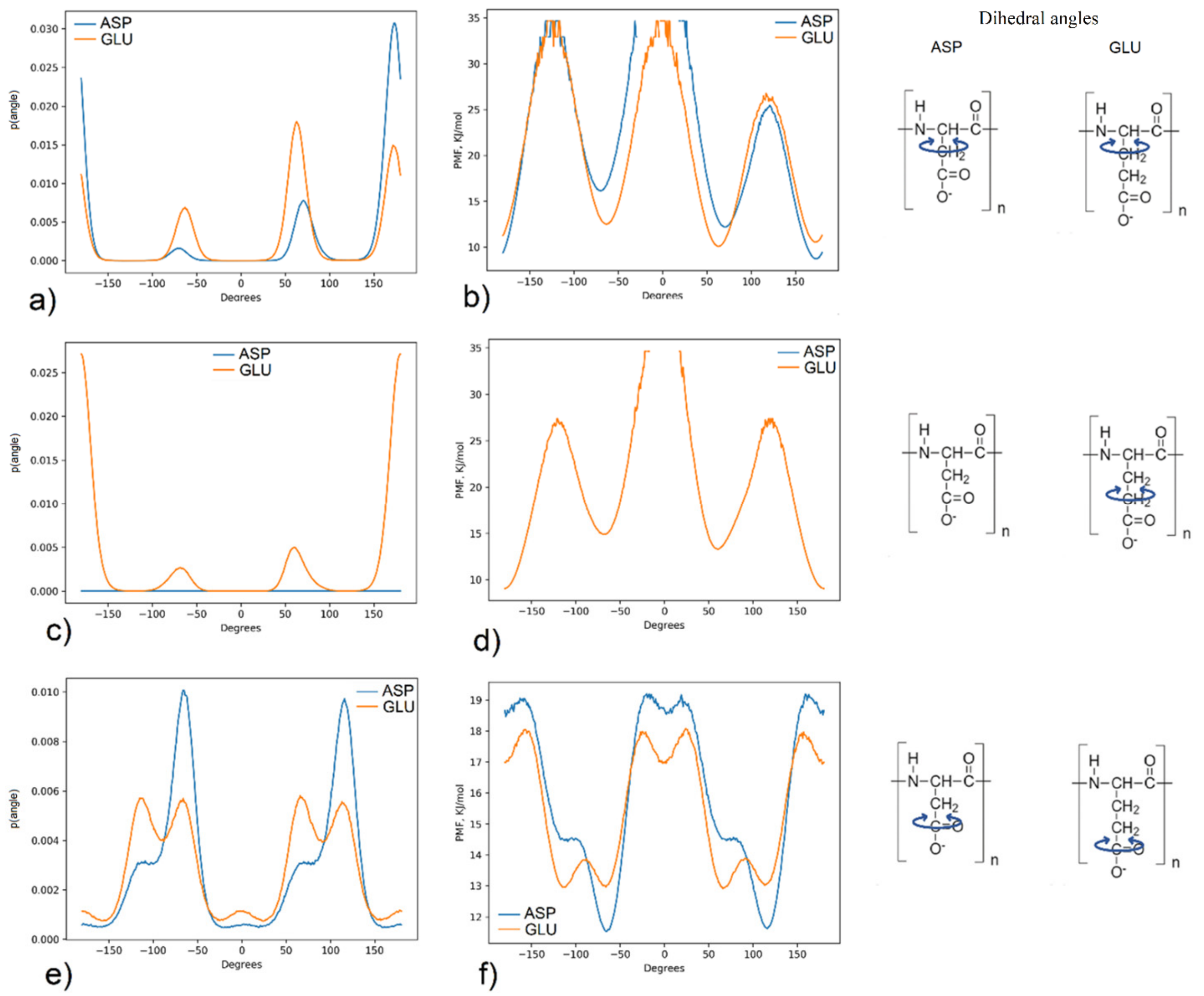

Figure S6. Dihedral angle distributions of the side chain (a,c,e) and potential mean force (b,d,f) obtained from the distributions with the schemes illustrating considering angles.

The distributions was obtained using 1 μs trajectory of the poly(amino acids) in water with degree polymerization 32. The potential mean force was obtained from the distributions using the following dependence:

$$PMF(\alpha) = -kT \ln g(\alpha) \quad (2)$$

where α – is dihedral angle, g(α) – distribution of α, k- the Boltzmann constant, T- temperature,.

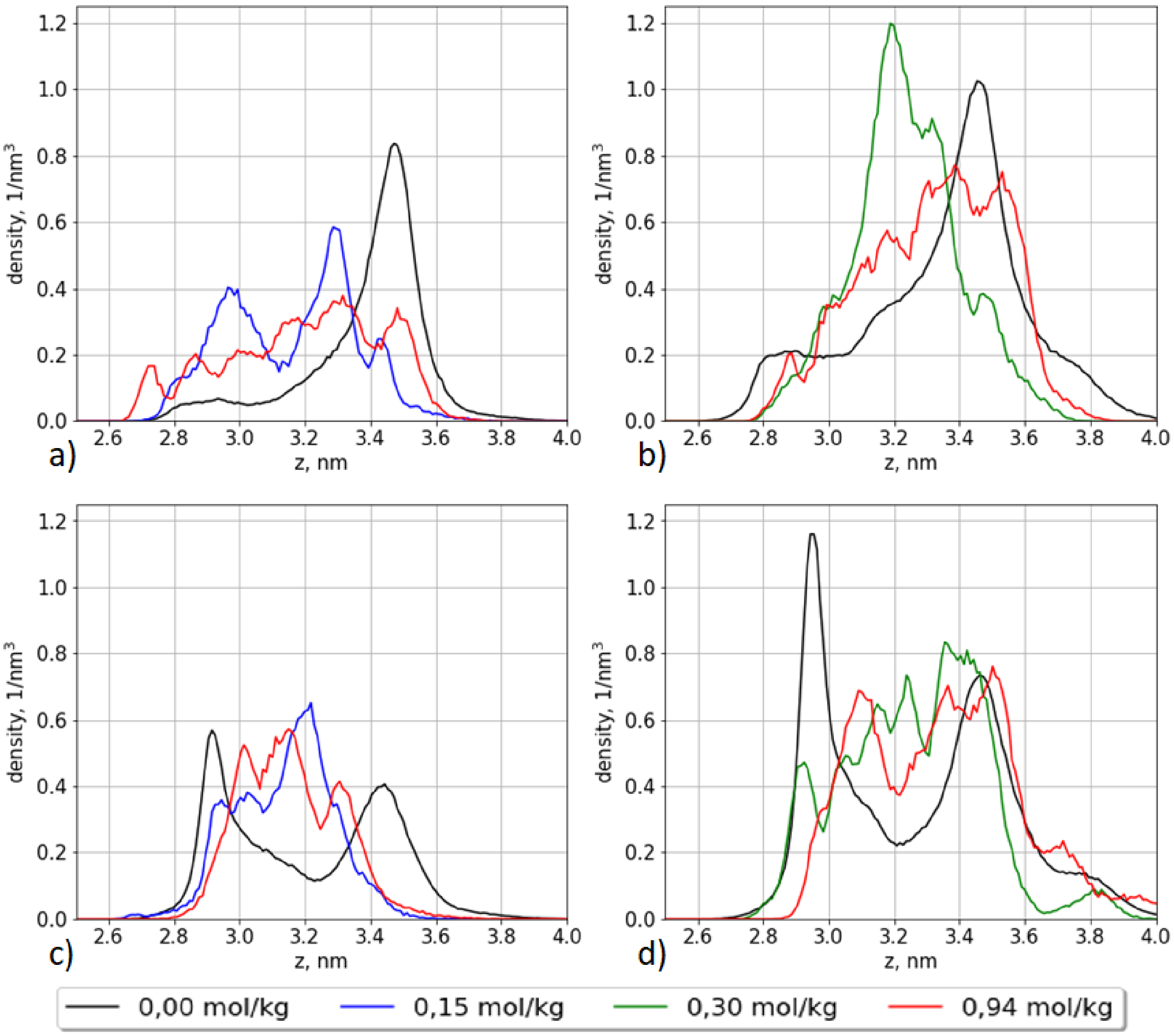

Figure S7. Density profiles of the ends of the grafted oligomers of aspartic and glutamic acids in at different $CaCl_2$ concentrations. a) Aspartic acid brush with 12% surface modification, b) aspartic acid brush with 25% degree surface modification, c) glutamic acid brush with 12% degree surface modification d) glutamic acid brush with 25% degree surface modification.
